# Popular Glucose Tracking Apps and Use of mHealth by Latinos With Diabetes: Review

**DOI:** 10.2196/mhealth.3986

**Published:** 2015-08-25

**Authors:** John Patrick Williams, Dirk Schroeder

**Affiliations:** ^1^ Hubert Department of Global Health Rollins School of Public Health Emory University Atlanta, GA United States; ^2^ Hubert Department of Global Health Rollins School of Public Health Emory University Marietta, GA United States

**Keywords:** diabetes mellitus, mobile health, mobile applications, systematic review, Hispanic

## Abstract

**Background:**

Diabetes mellitus in the United States is an increasingly common chronic disease, costing hundreds of billions of dollars and contributing to hundreds of thousands of deaths each year. The prevalence of diabetes is over 50% higher in Latinos than in the general population, and this group also suffers from higher rates of complications and diabetes-related mortality than NHWs. mHealth is a promising new treatment modality for diabetes, though few smartphone apps have been designed specifically for Latinos.

**Objective:**

The objectives of our study were: (1) to identify the most common features of the most popular diabetes apps and consider how such features may be improved to meet the needs of Latinos; (2) to determine the use of diabetes apps among a sample of online Hispanics in the US.

**Methods:**

Our study consisted of two parts. First, 20 of the most popular diabetes apps were reviewed in order to ascertain the most prevalent features and functionalities. Second, an online survey was fielded through a popular health website for Latinos (HolaDoctor) inquiring about respondents’ use of diabetes apps.

**Results:**

Approximately one-third of apps reviewed were available in Spanish. The most common features were blood glucose recording/annotation and activity logs. The majority of apps permitted exportation of data via e-mail but only a third enabled uploading to an online account. Twenty percent of apps reviewed could connect directly with a glucometer, and 30% had reminder functionalities prompting patients to take medications or check blood glucose levels. Over 1600 online surveys were completed during the second half of April 2014. More than 90% of respondents were from the United States, including Puerto Rico. The majority of respondents used a device running on an Android platform while only a quarter used an iPhone. Use of diabetes apps was approximately 3% among diabetic respondents and 3.6% among diabetic respondents who also had a smartphone. Among app users, blood glucose and medication diaries were the most frequently used functionalities while hemoglobin A1c and insulin diaries were the least used. A significant majority of app users did not share their progress on social media though many of these were willing to share it with their doctor.

**Conclusions:**

Latino diabetics have unique needs and this should be reflected in diabetes apps designed for this population. Existing research as well as our survey results suggest that many Latinos do not possess the prerequisite diabetes knowledge or self-awareness to fully benefit from the most prevalent functionalities offered by the most popular diabetes apps. We recommend developers incorporate more basic features such as diabetes education, reminders to check blood glucose levels or take medications, Spanish language interfaces, and glucometer connectivities, which are relatively underrepresented in the most popular diabetes apps currently available in Spanish.

## Introduction

### Diabetes Mellitus in the United States

Diabetes affects almost 26 million Americans—over 8% of the US population—and is the seventh leading cause of death in the United States [1]. Among Latinos, the proportion affected is approximately 11.8%, an almost 70% greater prevalence than in the general population [2]. According to the American Diabetes Association, the total cost of diabetes-related expenditures in 2012 was almost a quarter of a trillion dollars, translating to an average medical expenditure of almost $14,000 per patient [3]. A major source of these expenditures is hospitalizations, which have been shown to cost more for patients who have diabetes [4]. According to 2011 data from California where over a third of the population is Hispanic [5], the average cost of a hospitalization for a patient with diabetes exceeded that of a nondiabetic patient by over $2,000 [4]. Almost a third of all hospitalizations in California that year were for diabetic patients, a proportion which rose to over 40% among Latinos and was higher than that of African- or Asian-Americans [4].

Contributing to the problem is a lack of health literacy among Hispanics [6] which increases the risk of poor glycemic control [6]. Latinos have been shown to lag behind African-Americans and Whites in important health behaviors such as checking blood glucose levels, performing diabetic foot exams, and getting recommended vaccinations [7]. These findings are compounded by the feelings of many Latino patients that the care they receive from providers is frequently substandard [8] and problematic [9]. The number of Latino healthcare providers relative to the population is also small [10], and the resulting language challenges can lead to decreased patient compliance and worse outcomes [8]. Altogether, Latinos have worse glycemic control than the general diabetic population [11], are 60% more likely to start dialysis, and 50% more likely to die from diabetes than NHWs in the United States [12].

### The Potential for mHealth

mHealth is a promising new treatment modality for diabetic patients that has been shown in studies to improve glycemic control [2,13,14] It has also been found to be a potential source for cost savings and reduced burden on the health care system [15]. Though mHealth is broadly defined by the World Health Organization as “medical and public health practice supported by mobile devices” [16], the arrival of the smartphone in 2007 has caused an exponential proliferation of apps which have garnered increased attention among clinicians, researchers, and the federal government [17,18]. To date, there are few apps targeted specifically at Latinos with diabetes. This represents a missed opportunity, as over 90% of Latinos use a cell phone regularly - almost half of which are smartphones [19] - and they are just as likely as Whites to own a smartphone [20]. Given the challenges facing Latino diabetics with respect to health literacy and performance of health behaviors in the face of limited access to quality care, increased use of glucose tracking apps could facilitate reductions in poor outcomes in this population.

### Technology and the Latino Community

Evidence suggests that Latinos already have the capacity to use mobile technology to increase healthy behaviors. The Text4Baby Program, for example, involved the dissemination of text messages to pregnant women and mothers of newborns. A study by the National Latino Research Center revealed improvements in participants’ health knowledge, appointment attendance, and immunization adherence. Satisfaction with this program was also found to be higher among Spanish speakers [21]. The TExT-MED (Trial to Examine Text Messaging for Emergency Department patient with Diabetes) study in Los Angeles involved a similar intervention in which text messages were sent to low income inner-city patients with diabetes, almost 75% of whom were Latino. Results included improvements in healthy eating, increased physical activity, and higher medication adherence [22]. These studies suggest that simple interventions can be accepted and lead to improvements in healthy behaviors in Latinos, including those with diabetes.

Despite these encouraging findings, there is little research into the use of glucose tracking apps by Latinos or on which app functionalities are the most pertinent to this population. Recommendations endorsing specific apps for Latinos have been put forth by various organizations [23,24] though these are not research-based. There is therefore an unmet need for scholarly research into how mobile phone technology can best benefit Latinos suffering from diabetes.

### Goals of the Study

The goals of our study were twofold. First, we sought to identify the most prevalent functionalities of the most popular glucose tracking apps currently available. Second, we aimed to survey the usage of glucose tracking apps among Latinos visiting a popular Spanish language health website. In light of the challenges facing Latino diabetics, we attempted to set forth some basic guidelines for apps ideally suited to this population.

## Methods

### Overview

A systematic search strategy was used to select and review the most popular glucose tracking apps from official mobile phone stores. Each app was then examined and functionalities common to multiple apps were compiled into Table 1. A survey inquiring into glucose tracking app usage was then posted on the HolaDoctor website for a total of three weeks, during which time 1601 surveys were completed.

### Review of Glucose Tracking Apps

#### Overview

Searches for eligible apps on the iPhone and Android platforms were conducted On January 4^th^ (Apple) and 5^th^ (Android), 2014. A total of ten apps were selected from each platform (five free and five paid). iPhone apps were selected by navigating to the Medical section of Apple’s App Store and clicking the link “View Medical in iTunes” in the upper right hand corner. This required previous installation of iTunes on the computer’s hard drive [25]. Android apps were selected in a similar fashion by locating the list of the top free and paid medical apps on the Google Play website [26]. The first five apps in each section meeting eligibility criteria were selected. Because some apps had versions available on both iPhone and Android platforms or had both free and paid versions listed in the search results, several of the selected apps had multiple versions reviewed. The authors felt this would not be redundant, however, as features of such apps were found to vary depending on platform and cost.

#### Eligibility Criteria

Apps were considered eligible if they could be found in the “medical” section of the Apple or Google Play stores and had the capacity to record and recall blood glucose measurements. All apps selected for review possessed additional functionalities (see Multimedia Appendix 1), though the quantity and characteristics of these were not considered during the selection process.

#### Data Extraction

**Figure 3:**
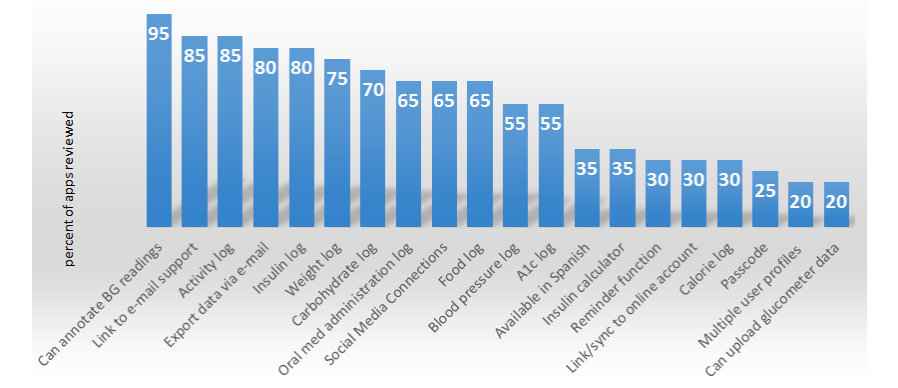
Prevalence of functionalities found in selected glucose tracking apps.

Reviews of all iPhone apps with the exception of Track3 by Coheso, Inc. were carried out on an iPod Touch running iOS version 6.1.5. Evaluation of the Track 3 app required a more recent version of iOS, thus an iPhone 4S running iOS version 7.0.4 was used. Reviews of Android apps were carried out with either a Nexus 4 running Android version 4.4.2 or a laptop running BlueStacks App Player for Windows (beta-1). App functionalities for all apps were investigated by author JW (see Figure 3). Product descriptions from the Apple App Store, Google Play website, or app developer websites were referenced as needed to clarify uncertainties. Apps were classified as being available in Spanish if either a change in the language setting of the mobile phone device from English to Spanish resulted in a meaningful change in the language of the app display or if the app itself had a language setting that included Spanish. Prices listed for each app were current as of January 4 (Apple App Store) or January 5 (Google Play website), 2014.

### Online Survey

An online survey was posted on the HolaDoctor website [1] from April 15 to May 1, 2014 (see Multimedia Appendix 1). HolaDoctor’s website, also available through Univision.com as *Univision Salud con HolaDoctor*, is the most frequently visited Spanish language health website on the Internet, with over 3.5 million monthly unique visitors, of which over 1 million reside in the US. Over half of HolaDoctor’s traffic access the website through mobile devices. The survey was available only in Spanish. Questions explored respondents’ diabetes status as well as use of mobile phones and glucose tracking apps. A total of 1161 surveys were completed over an initial 17 day period. After several user comments reported unfamiliarity with the term “app,” a second survey using clarifying language was posted from May 12 to May 19, 2014. This resulted in the completion of an additional 440 surveys for a total of 1601 surveys. Summary data can be found in Table 1.

## Results

### Review of Glucose Tracking Apps

#### Overview

**Figure 1:**
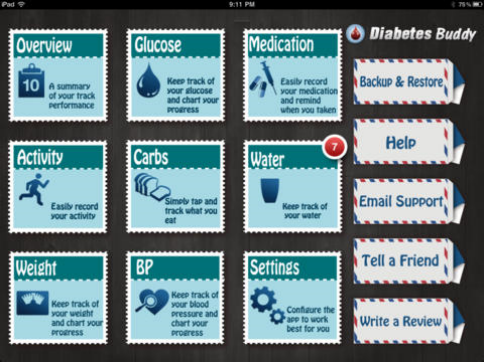
Screenshot of Diabetes App Lite by BHI Technologies, Inc. https://itunes.apple.com/us/app/diabetes-app-lite-blood-sugar/id387337850?mt=8.

**Figure 2:**
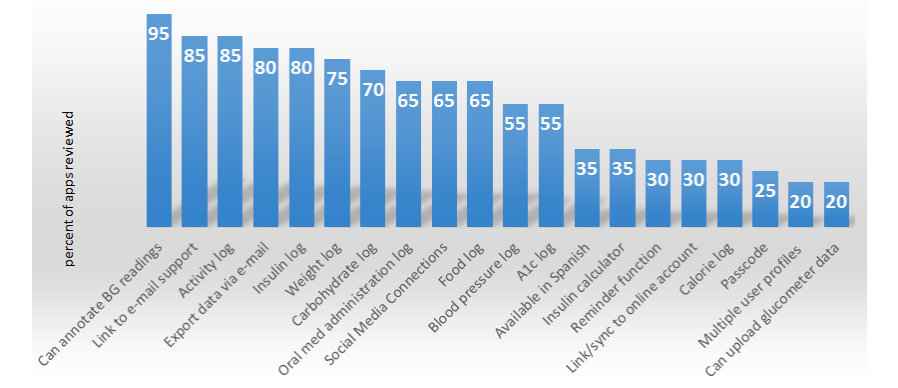
Prevalence of functionalities found in selected glucose tracking apps.

Samples of the results of the app review can be found in Figure 1, Figure 2, and in Multimedia Appendix 1. A total of 20 apps were reviewed, though some apps were reviewed multiple times as described in the Methods section. It should be noted that while the ability to record and recall blood glucose measurements was the primary selection criterion, analytical capabilities such as calculations of averages and creations of figures including graphs and flow sheets invariably accompanied this functionality. Thirty five percent of all apps reviewed were available in Spanish (20% of iPhone apps and 50% of Android apps). The ability to annotate blood glucose readings was the most common feature, while pass codes and the capacity for multiple user profiles were the least common. Only 20% of apps could download data directly from glucometers.

#### Price

The price of apps ranged from free up to ten dollars with the average price of paid apps being approximately $5.03. The average price for iPhone apps ($6.39) was higher than that for Android apps ($3.66).

#### Documentation Functionalities

Activity logs (85%) were the most prevalent documentation functionality followed by insulin logs (80%), weight logs (75%), and carbohydrate logs (70%). While 80% of the apps reviewed included insulin administration logs, only 65% included logs for oral or injectable noninsulin medications. Carbohydrate and food logs (70% and 65%, respectively) were featured more often than calorie logs (30%).

#### Information Sharing

Data export via e-mail was present on 80% of apps while social media connections were featured on 65%. Fewer than a third of apps allowed users to upload their data to online app-sponsored accounts.

#### Glucometer Connectivity

Twenty percent of apps permitted download of blood glucose measurements from a glucometer. The prevalence of this functionality was equal for both Apple and Android apps.

### Online Survey

#### Diabetes Demographics

Table 1 shows the results of the online survey. A total of 1601 surveys were completed. 68.9% (1103/1601) of responses came from the continental United States and 22.0% (353/1601) came from Puerto Rico. 2.9% (46/1601) were from Mexico, while 3.4% of respondents (54/1601) marked “unknown.” 36.7% of respondents (588/1601) reported a history of diabetes, and 17.2% (276/1601) reported caring for a family member with the disease. Among those who reported a personal history of diabetes, nearly 13% (74/588) reported having type I, while 69% (408/588) reported having type II, and 18% did not know which type of diabetes they had. Among those reporting type I diabetes, fewer than half (29/74) reported taking insulin.

**Table 1:** Survey results, all respondents (n=1601).

General characteristics		Number (%)
**Country**		
	United States	1103 (68.89)
	Puerto Rico	353 (22.05)
	Mexico	46 (2.87)
	Other^a^	45 (2.81)
	Unknown	54 (3.37)
**Do you have diabetes?**		
	Yes	588 (36.73)
	No	491 (30.67)
	I don’t know	246 (15.37)
	I take care of a family member with diabetes	276 (17.24)
**Mobile phone platform**		
	Android	815 (50.91)
	iOS	415 (25.92)
	Blackberry	17 (1.06)
	Do not have mobile phone	354 (22.11)

^a^Countries in this category included Argentina, Brazil, Canada, Chile, Colombia, Costa Rica, the Dominican Republic, Ecuador, El Salvador, Guatemala, Nicaragua, Panama, Peru, Spain, Switzerland, and Venezuela

#### Mobile Phone Usage and Glucose Tracking App Usage

77.8% of respondents (1,247/1601) reported using a mobile phone. Approximately 65% (815/1247) of these used Android devices while 33.3% (415/1247) used an iPhone; seventeen respondents used a Blackberry. Roughly 2% (33/1601) of all respondents reported using a glucose tracking app; this increased to 3% (18/588) among diabetics and 3.6% (16/449) among respondents with both a history of diabetes and mobile phone use. Among diabetics who used apps, about half (n=10) used them in Spanish while about a quarter (n=4) used them in English. Almost a quarter of respondents reported not knowing in which language they used the app.

#### Cost

Nearly half of glucose tracking app users downloaded free apps. This proportion increased to 61.5% (8/13) when excluding respondents unable to recall the price of their app. Conversely, 38.4% of respondents (5/13) able to recall the price of the app paid money for it. Of these five respondents, three of them paid three dollars or more.

**Table 2:** Survey results among patients reporting a history of diabetes (n=588).

General characteristics		Number (%)
**Country**		
	United States	415 (70.6)
	Puerto Rico	133 (22.6)
	Mexico	15 (2.6)
	Other^a^	8 (1.4)
	Unknown	17 (2.9)
**Diabetes type**		
	Type I	74 (12.6)
	Type II	408 (69.4)
	Don’t know	106 (18.0)
**Do you use insulin?**		
	Yes	161 (27.4)
	No	427 (72.6)
**Do you use insulin? (type I only, n=74)**		
	Yes	29 (39.2)
	No	45 (60.8)
**Do you use insulin? (type II only, n=408)**		
	Yes	111 (27.2)
	No	297 (72.8)
**Do you use a diabetes app?**		
	Yes	18 (3.1)
	No	570 (96.9)

^a^Countries in this category included Argentina, Brazil, Canada, Chile, Colombia, Costa Rica, the Dominican Republic, Ecuador, El Salvador, Guatemala, Nicaragua, Panama, Peru, Spain, Switzerland, and Venezuela

#### Documentation and Reminder Functionalities

Tracking of oral medications was the most popular documentation functionality, with 50% of respondents expressing approval (9/18), followed by 44% endorsing blood glucose monitoring (8/18). Exercise tracking, with 22% endorsement (4/18) was featured less often than dietary monitoring of carbohydrates or calories consumed, at 33% (6/18). Insulin and A1c tracking were the least commonly utilized documentation functionalities, at 17% each (3/18). 50% and 44% of respondents (9/19 and 8/18 respectively) reported frequent use of reminders to check blood glucose or take medications.

#### Information Sharing

The majority of respondents (83%) either kept their data private or shared it only with their doctor. The remaining 17% shared information about their diabetes on social media outlets such as Facebook, Twitter, or diabetes forums.

**Table 3:** Characteristics of app usage among diabetic respondents reporting use of diabetes apps (n=18).

General characteristics		Number (%)
**Language in which app is used**		
	English	4 (22)
	Spanish	10 (56)
	I don’t know	4 (22)
**How much did you pay for the app?**		
	Free	8 (44)
	$0.99	1 (6)
	$2.99	1 (6)
	More than $3.00	3 (17)
	I don’t remember	5 (28)
**Proportion of respondents reporting frequent use of the following documentation functionalities**		
	Oral medications	9 (50)
	Blood glucose	8 (44)
	Blood pressure	6 (33)
	Diet-related	6 (33)
	Weight	5 (28)
	Exercise	4 (22)
	A1c	3 (17)
	Insulin	3 (17)
	None of these	4 (22)
**Proportion of respondents reporting frequent use of the following reminder features**		
	Reminder to check blood glucose	9 (50)
	Reminder to take medications	8 (44)
	None	4 (22)
**Information sharing**		
	Shares with physician only	10 (56)
	Does not share with anyone	5 (28)
	Diabetes forums	2 (11)
	Facebook	1 (6)
	Twitter	1 (6)

## Discussion

### Summary of Study

In this study, we set out to characterize the most prevalent functionalities for popular glucose tracking apps and survey Latinos on their use of these apps. We went about this task by selecting and reviewing 20 of the most popular glucose tracking apps on the market as of January, 2014 and posting an online survey on one of the most popular Spanish language health websites on the Internet. In our app review we found blood glucose analytical instruments (eg graphs, flow sheets, statistics) to be the most prevalent functionalities. These were frequently accompanied by documentation of dietary and biometric data as well as functionalities enabling users to share data on social media. In contrast, a minority of apps were available in Spanish, contained reminder functionalities encouraging adherence to blood glucose monitoring and medication regimens, or allowed download of data directly from glucometers.

Our online survey found that approximately three percent of respondents with diabetes used a glucose tracking app, a proportion that is higher than the estimated global average of 1.2% [27]. Of these, the number of respondents running their apps on Android products was nearly double the number of those running their apps on Apple products. Most of the apps used were free to download. Fifteen percent of respondents reported not knowing their diabetes status and fewer than half of self-reported type I diabetics reported using insulin. These findings support the findings from other studies [6,28-30] that there is a lack of diabetes knowledge and awareness among Latinos.

### Comparison With the Literature

At least three studies have been completed on the prevalence of various glucose tracking app functionalities using systematic search strategies [31-33]. Two of the studies reviewed apps limited to one platform. The 2011 article by Chomutare et al [31] is the only study of the three to consider both major platforms, reviewing 49 iPhone apps and 33 Android apps. In comparison with this 2011 review, we found a significantly greater prevalence of multiple functionalities including medication management, diet management, physical activity monitoring, and disease-related reminders. The starkest contrast between the earlier review and ours was the prevalence of social media. Only 15% of apps had social media functionalities in the 2011 review versus 65% of apps in our review. This contrast most likely reflects the growing role of social media in daily life [34].

A limited number of studies have evaluated glucose tracking app use in a specific segment of the population. An article by Arnhold et al [35] studied the usability of glucose tracking apps among patients ages 50 years and older. Our study, while not providing empirical evidence as to which app functionalities work best for a specific subgroup, agrees with Arnhold et al’s finding that there is a need for further investigation into how mobile phone apps can be tailored to specific target audiences.

### How Glucose Tracking Apps Designed for Latinos Should be Different

#### Overview

Latinos lag behind NHWs in levels of health literacy, which in turn has been shown to affect glycemic control [6]. This population also tends to have worse self-management practices [36], including lower levels of physical activity and inconsistent self-monitoring of blood glucose [37]. Glucose tracking apps should address these disparities in knowledge and practice using carefully selected functionalities tailored to this population.

#### Education

Education has been shown to be an underrepresented feature of most glucose tracking apps [31] but should be included in apps for Latinos considering both the lower overall levels of educational attainment of Latinos relative to NHWs [38] as well as the findings of our survey. Content should be provided at a basic reading level and available in both English and Spanish. In addition, audio or video-based educational materials could complement text, as they may help bypass literacy barriers and would likely be well accepted among Latinos who are already major consumers of online multimedia [19].

#### Self-Management Functionalities

Self-management functionalities focusing on blood glucose monitoring, diet, and exercise should be easy to use and motivational. The number of functionalities on a single app should be kept to the minimum necessary to encourage consistency without decreasing usability [32]. Self-management practices can be encouraged by minimizing the burden of data entry and by employing reminders (eg to check blood glucose or take medication) to minimize unintentional nonadherence [39,40].

#### Data Entry Burden

Data entry burden can be reduced through the use of simplified graphic interfaces with adjustable text and icon sizes for elderly or visually impaired users [41] as well as glucometer connectivity. Glucometer connectivity is currently lacking in most popular apps according to our study which found that only 20% of the apps reviewed had this capacity.

#### Reminders

Reminder functionalities were available in fewer than third of the apps reviewed, though our survey found that the majority of app users surveyed used reminders regularly. Automated reminders can serve several functions, not the least of which could include boosting medication adherence and self-monitoring of blood glucose. Periodic reminders for feet exams, physician visits, and yearly flu vaccines can also be incorporated. Besides conveying instructions, reminders can be educational and/or motivational in a manner similar to the text message interventions used in the Text4Baby and TExT-MED programs. App content should always be culturally appropriate [42] and mindful of social determinants of health as well as the social and cultural heterogeneity within the Latino population itself [43].

### Limitations of the Study

Our app review included only a small fraction of the glucose tracking apps available for download on the iTunes app store and Google Play. Methods used to select apps were subject to proprietary ranking algorithms by Apple and Google and thus the apps reviewed may not represent those of the highest quality as judged by more impartial measures such as third party ratings. To these authors’ knowledge, however, no such rating system exists for glucose tracking apps. Nevertheless, there may have been apps of high quality that were not reviewed.

For the survey portion of the study, respondents on the HolaDoctor website constituted a convenience sample which may not reflect the entire Hispanic population. With its very large visitor base of over 1 million monthly unique users, the HolaDoctor website does, however, fairly represent the *online* Hispanics in the United States. Furthermore, evidence suggests that the majority of Latinos in the United States already use the internet in some fashion [44]. Nevertheless, this does largely exclude the elderly, those with less than a high school education, and those who are predominantly Spanish speaking [44], groups who shoulder a significant proportion of the diabetes burden within the Latino population as a whole. Given that 17% of the respondents in our survey reported providing care for a family member with diabetes, however, it is possible that the benefits of diabetes apps may extend beyond the immediate user to family members, specifically the elderly. Respondents living in countries other than the United States were also permitted to complete surveys, and this may affect the generalizability of the study’s conclusions given the inherent variation in social, cultural, and economic conditions between countries. The effect of this variation is likely to be minor, however, as the vast majority of responses (93%) came from the continental United States or Puerto Rico.

Finally, a number of the online surveys contained internally inconsistent responses. In particular, fewer than half of type I diabetics reported using insulin. Such incongruous responses may be due to respondents’ confusion with the survey questions, though the authors suspect it stems more from a lack of knowledge and awareness regarding what type of diabetes they have, if any.

### Conclusion

There is a significant need for diabetes apps targeted at Latinos. Given the high prevalence of diabetes in the Latino population and the CDC estimating a 50% lifetime diabetes risk for Latino children born in the year 2000 [45], apps designed specifically for this population will be needed to realize the full potential of mHealth to improve the lives of those affected by this disease.

## Appendix

Multimedia Appendix 1 Review of selected glucose tracking apps.

## Abbreviations

A1c: glycosylated hemoglobin
iOS: iPhone operating system
mHealth: mobile health
NHW: non-Hispanic White
TExT-MED: trial to examine text messaging for emergency department patient with diabetes
