# Supplementary figures and images for: Popular Glucose Tracking Apps and Use of mHealth by Latinos With Diabetes: Review
Source: JMIR Mhealth Uhealth. 2015 Aug 25;3(3):e84. doi: 10.2196/mhealth.3986 (PMC4705030; doi:10.2196/mhealth.3986)

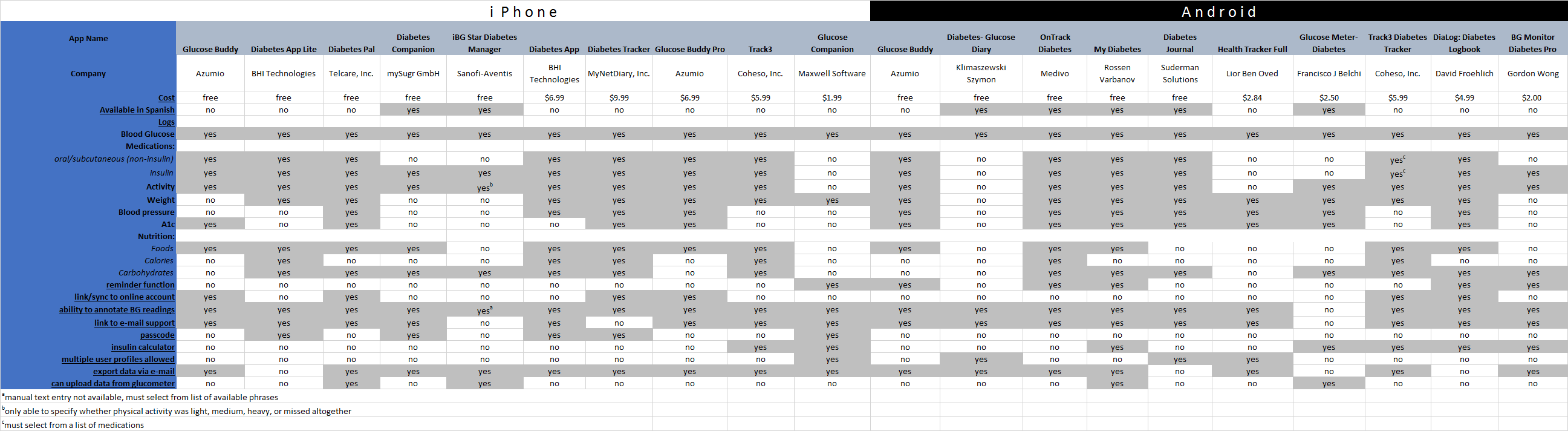

Supplement: Multimedia Appendix 1 [file mhealth_v3i3e84_app1.png]
